# Extranodal spread of primary and secondary metastatic nodes: The dominant risk factor of survival in patients with head and neck squamous cell carcinoma

**DOI:** 10.1371/journal.pone.0183611

**Published:** 2017-08-24

**Authors:** Misa Sumi, Shuntaro Sato, Takashi Nakamura

**Affiliations:** 1 Department of Radiology and Cancer Biology, Nagasaki University Graduate School of Biomedical Sciences, Nagasaki, Japan; 2 Clinical Research Center, Nagasaki University Hospital, Nagasaki, Japan; Technische Universitat Munchen, GERMANY

## Abstract

Extranodal spread (ENS) in patients with head and neck squamous cell carcinoma (HNSCC) can greatly influence the prognostic outcomes. However, the relative risks of ENS in the primary (1st) and secondary (2nd) metastatic nodes (mets) are not well documented. We retrospectively analyzed the hazard ratios (HRs) of ENS in the 1st and 2nd mets from 516 HNSCC patients who had undergone primary tumor excision. The impact of clinically and/or histologically confirmed ENS-positive mets on prognosis in terms of cancer-specific survival was analyzed. Cox proportional hazard regression analysis indicated that ENS-positive 1st met (adjusted HR = 3.15; 95% CI, 1.40–7.56; p = 0.006) and ENS-positive 2nd met (adjusted HR = 4.03; 95% CI, 1.41–16.96; p = 0.007) significantly and independently predicted poor prognosis; however, other variables including primary site, met size or numbers, and met location in the contralateral side of the primary lesion, did not. Cumulative incidence function and Cox analyses indicated that differences in ENS profiles of 1st and 2nd mets stratified HNSCC patients with varying risks of poor outcome; HRs relative to patients with ENS-positive 1st met (-)/ENS-positive 2nd met (-) were 4.02 (95% CI, 1.78–8.24; p = 0.002), 8.29 (95% CI, 4.58–14.76; p <0.001), and 25.80 (95% CI, 10.15–57.69; p <0.001) for patients with ENS-positive 1st met (+)/ENS-positive 2nd met (-), ENS-positive 1st met (-)/ENS-positive 2nd met (+), and ENS-positive 1st met (+)/ENS-positive 2nd met (+) patients, respectively. Kaplan-Meier analysis indicated that the 2nd met that appeared in the neck side with a history of 1st met and neck dissection had a higher risk of ENS than the 2nd met in the neck side without the history (p = 0.003). These results suggested that ENS is a dominant prognostic predictor of HNSCC patients, with double-positive ENS in the 1st and 2nd mets predicting the most devastating outcome.

## Introduction

Metastasis to the regional lymph node has been considered a critical risk factor in patients with head and neck squamous cell carcinoma (HNSCC). A metastatic node (met) in the neck has been found to be closely related to the prognosis of HNSCC patients [1]. However, the causal relationship between lymph node metastasis and poor prognosis of HNSCC patients has not been well understood due to significant contributions of other demographic and clinical factors to the prognostic outcomes of patients. Patient prognosis is further deteriorated by extensions of metastatic cancer cells beyond the nodal capsule (extranodal spread, ENS), which is frequently associated with high rate of locoregional and distant failures [2–5].

The development of secondary (2nd) met may occur after surgical excision of the primary lesions without any locoregional failure of the primary site or distant metastasis, which could further deteriorate the prognosis of patients. Also in such cases, the ENS would be an additional prognostic risk factor. Neck dissection (ND) may be performed for the primary (1st) met(s) that was histologically confirmed on biopsy specimens and/or was suspected based on imaging information without histological evidence. The lymphatic systems in the neck and the microenvironment of the regional lymph nodes may be altered after surgical interventions for the primary and neck lesions [6, 7], which might facilitate cancer metastasis to regional lymph nodes and the development of ENS in the 2nd mets.

Therefore, it is very interesting to know the extent to which the neck disease-related factors, including 1st/2nd mets, ENS in the 1st/2nd mets, and ND can contribute to the prognosis of HNSCC patients who had undergone surgical excision of the primary lesions. Accordingly, in the present study, we retrospectively investigated the impact of ENS in the 1st and/or 2nd mets on the prognosis of HNSCC patients in terms of cancer-specific survival, by assessing the relative risk factors of the different profiles of 1st/2nd mets and ENS. It should be noted that, by including the 2nd met for analysis in this study, we excluded patients who developed locoregional failures in the primary site and/or distant metastases during the periods from the surgery up to the development of 2nd met, which did not fulfill the requirements of 2nd met. Thus, the risks obtained in the present study were minimally influenced by post-treatment conditions of the primary and distant metastatic lesions.

## Materials and methods

### Data collection

**Fig 1:**
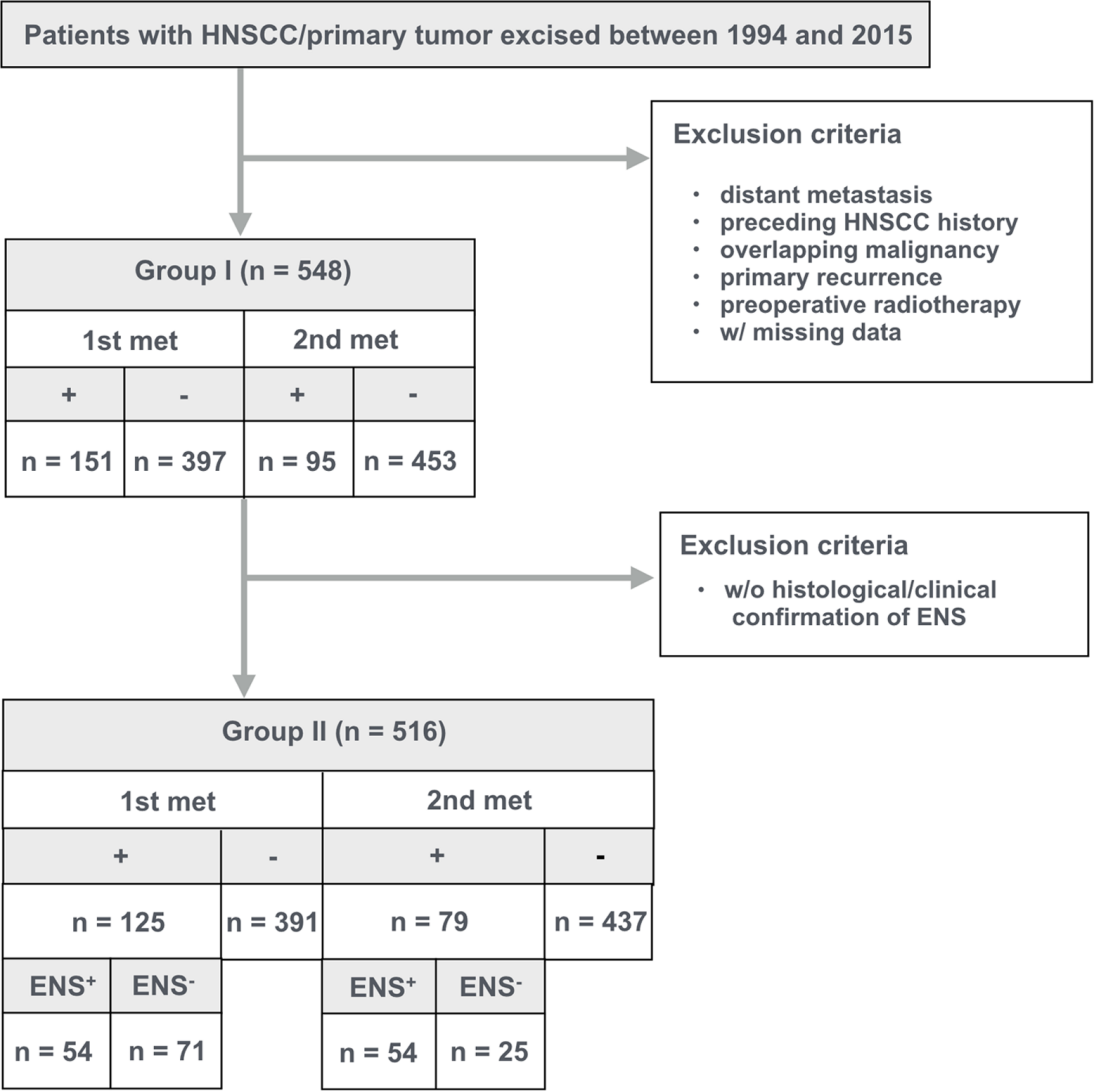
Consort diagram shows definition and metastatic node (met) profiles of 2 patient groups (Group I and II). Group I patients were selected from the clinical data base as those who underwent surgical excision of primary HNSCC between 1994 and 2015, were examined preoperatively and postoperatively with CT or MR imaging for the primary and neck lesions, and did not fit the exclusion criteria listed (78 of 598 had died from cancer at analysis); and Group II patients were from the Group I as those who were histopatholocally or clinically confirmed to have or have not ENS (63 of 516 had died from cancer at analysis).

We searched the clinical databases of the Nagasaki University Hospital and identified 548 patients (373 men and 175 women; median age, 67 years; age range, 26–95 years) with HNSCC who underwent excision of the primary tumor with or without ND [ND (+), n = 316 and ND (-), n = 232], and were examined preoperatively and postoperatively with computed tomography (CT) or magnetic resonance (MR) imaging for the primary and neck (metastatic node) lesions between 1994 and 2015 (Group I, **Fig 1**). Patients who had distant metastasis, preceding HNSCC history, overlapping malignancy in other parts of the body, or recurrence of the primary lesion, or had received pre-operative radiotherapy were excluded from the study cohort. The study protocol was approved by the ethics committee of Nagasaki University Hospital, and the requirement to obtain informed consent for the review of images and records was waved.

Primary metastatic [1st met (+), n = 151] or non-metastatic [1st met (-), n = 165] nodes were confirmed on the pathology report of the excised ND specimens. ND (-) patients (n = 232) were categorized in the 1st met (-) patient group. The secondary metastasis (2nd met) was defined as a metastatic node that occurred after the initial surgical interventions for the primary tumor without any preceding primary recurrence or distant metastasis during the follow-up period, and that was histologically and/or clinically confirmed. The median duration between excision of the primary tumors and the diagnosis of a 2nd met was 6 months (range, 1–61 months). The median follow-up period of the 548 patients was 54 months (1–241 months).

ENS-positive nodes of the 1st met were histologically (n = 54) diagnosed, and those of the 2nd met were histologically (n = 38) or clinically (n = 16, based on CT or MR imaging) diagnosed in 516 (346 men and 170 women; median age, 67 years; age range, 26–95 years) of the 548 HNSCC patients (Group II, **Fig 1**).

The primary sites of the Group I/Group II patients included the tongue (n = 180/n = 172), lower gingiva (81/75), oral floor (50/46), hypopharynx (48/42), larynx (46/45), upper gingiva (42/40), oropharynx (35/31), buccal mucosa (24/24), ear (12/12), maxillary sinus (9/9), palate (9/8), lip (6/6), upper plus lower gingiva (4/4), and nasal cavity (2/2). The summary statistics of the 548 (Group I) and 516 (Group II) patients are shown in **Table 1**.

**Table 1:** Demographic and clinical statistics of Group I (n = 548) and Group II (n = 516) HNSCC patients.

	Group I (n = 548)	Group II (n = 516)
age		
distribution (years)		
≤29	3	3
30–49	57	57
50–69	257	237
70–89	226	214
≥90	5	5
sex		
men: women	373: 175	346: 170
primary site		
oral cavity	396	375
hypopharynx	48	42
larynx	46	45
oropharynx	35	31
miscellaneous	23	23
1st met		
(-)	397	391
(+)	151	125
1st met profiles in the neck (size/no./location) §		
≤3 cm/single/ipsilateral	—	44
>3 cm and ≤6 cm/single/ipsilateral	—	4
≤6 cm/multiple/ipsilateral	—	52
≤6 cm/single or multiple/contralateral	—	20
>6 cm	—	5
ENS-positive 1st met		
(-)	—	462
(+)	—	54
no. of ENS-positive 1st met (single/multiple)	—	39/15
2nd met		
(-)	453	437
(+)	95	79
no. of 2nd met (single/multiple)		53/26
ENS-positive 2nd met		
(-)	—	462
(+)	—	54
no. of ENS-positive 2nd met (single/multiple)		44/10
ND		
(-)	232	227
(+)	316	289
causes of death		
head and neck SCCs	78	63
other causes ¶	15	14
cancer in other organs	5	5
GI tract cancer	2	2
lung cancer	1	1
uterus cancer	1	1
bladder cancer	1	1
suicide	2	2
myocardial infarction	1	1
asthma	1	1
brain aneurysm	1	0
senility	1	1
unknown	4	4

§, Nodal size was determined on axial CT or MR images and was expressed in the maximum diameter; location was referred as the neckside relative to the primary lesion (ipsilateral or contralateral).

¶, The primary and neck lesions were controlled and any distant metastasis was not evident in these patients.

### Imaging criteria for 2nd met and ENS

The 2nd met was identified based on histological results from biopsy specimen or specimen from the neck dissection for 2nd met, or on CT and/or MR imaging findings. A 2nd met with or without ENS was identified on CT and/or MR images as a nodal structure in the neck that fulfilled at least one of the following criteria: (a) non-enhanced focal defects in the nodal parenchyma, (b) apparent size-up on images during follow-up periods, or (c) imaging characteristics of ENS. An ENS-positive node was defined on CT and/or MR images as a nodal structure having irregular or interrupted enhancement at the perimeter on contrast-enhanced CT and/or MR images.

### Statistical analysis

Cancer-specific survival of the patients was estimated using cumulative incidence function in the presence of competing risk such as death due to non-SCC tumor or non-cancer deaths (**Table 1**). Cumulative incidences in different patient groups were compared using Gray’s test. The Bonferroni correction was used to adjust p-values in multiple comparisons.

Univariate and multivariable Cox proportional hazard regression analyses were used to test for independent significance with varying combinations of the following parameters included in the model as covariates: sex, age in grade, primary site, the presence or absence of 1st met, the presence or absence of 2nd met, the presence or absence of ENS in the 1st met, the presence or absence of ENS in the 2nd met, and the number of ENS-positive 1st or 2nd mets. Variables with p values of <0.05 at the determination of unadjusted HRs were incorporated in multivariable analysis to assess the adjusted HRs. The relative HRs of varying combinations of met variables were calculated, with a particular set of combined met variables used as a reference (HR = 1). The Cox proportional hazard regression analysis was performed on the variables that were confirmed not to violate the proportional hazards (PH) assumption. Schoenfeld residuals were used for assessing the violation of PH assumption. We found that the PH assumption was not violated in all models.

Influence of pre-existing 1st met and history of ND in the same neck side of 2nd met on cancer specific survival was estimated using Kaplan-Meier analysis, and log-rank test was applied to assess the significance in cancer-specific survival rates between groups.

Cox proportional hazard regression analysis, and Kaplan-Meier analysis with log-rank test were performed using JMP Pro (SAS, version 13) software. R software version 3.4.0 (2017-04-21, https://www.R-project.org/) was used for assessing PH violations and Cumulative incidence function.

## Results

### Prognostic importance of 1st and 2nd met

We first analyzed the prognostic importance of demographical and neck disease profiles of the 548 patients with HNSCC. Multivariable Cox proportional hazard regression analysis indicated that 1st or 2nd mets were significant and independent risk factors in HNSCC patients [adjusted HR = 2.00; 95% CI, 1.17–3.53; p = 0.01 for 1st met (+) patients, and 4.55; 95% CI, 2.85–7.21; p <0.001, for 2nd met (+)].; however, age, sex, primary site, and the history of ND were not (**Table 2**).

**Table 2:** Cox proportional hazard regression analysis for important prognostic factors in 548 HNSCC patients (Group I).

	n	hazards ratio	95% CI	p-value
univariate analysis				
age in grade (per 20 years)		0.90	0.66–1.25	0.54
≤29	3			
30–49	57			
50–69	257			
70–89	226			
≥90	5			
gender (men vs. women)	373/175	2.16	1.25–4.01	0.005
primary site				
hypopharynx vs. larynx	48/46	4.04	1.45–14.25	0.007
hypopharynx vs. oropharynx	48/35	3.55	1.16–15.41	0.02
hypopharynx vs. oral cavity	48/396	2.29	1.22–4.00	0.01
hypopharynx vs. miscellaneous	48/23	2.90	0.95–12.60	0.06
hypopharynx vs. others	48/500	2.49	1.34–4.30	0.005
1st met (+) vs. 1st met (-)	151/397	3.29	2.11–5.16	<0.001
2nd met (+) vs. 2nd met (-)	95/453	4.59	2.92–7.16	<0.001
ND (+) vs. ND (-)	316/232	2.50	1.51–4.35	<0.001
multivariable analysis				
gender (men vs. women)	373/175	1.43	0.81–2.72	0.23
primary site (hypopharynx vs. others)	48/500	1.90	0.996–3.64	0.051
1st met (+) vs. 1st met (-)	151/397	2.00	1.17–3.53	0.01
2nd met (+) vs. 2nd met (-)	95/453	4.55	2.85–7.21	<0.001
ND (+) vs. ND (-)	316/232	1.59	0.82–3.10	0.17

HNSCC, head and neck squamous cell carcinoma; 1st met, primary metastasis; 2nd met, secondary metastasis; ENS, extranodal spread; 95% CI, 95% confidence interval, ND, neck dissection. Patients were categorized into either of the age groups of ≤29, 30–49, 50–69, 70–89, or ≥90 years. In multivariable analysis, adjusted hazards ratios were estimated by incorporating variables that were significant in univariate analysis. n, numbers of patients.

**Fig 2:**
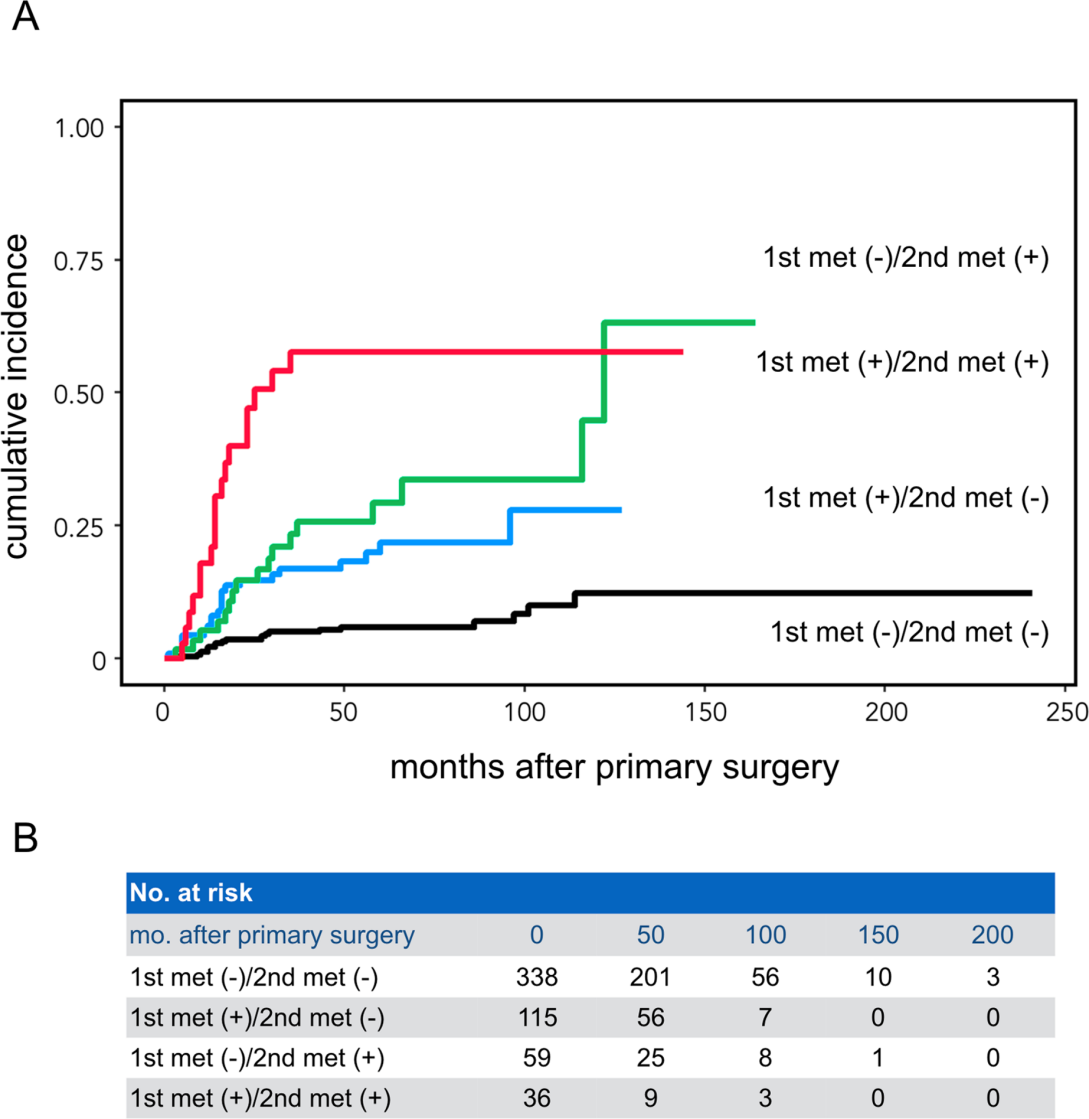
Cumulative incidence function in 548 HNSCC patients with different 1st and 2nd met profiles. The number of cases at risk (no. at risk) at each time point (0–200 months) was provided in B. Gray’s test was used to compare cumulative incidences between different groups. The Bonferroni correction was used to adjust p-values in multiple comparisons in the Gray’s test and a difference in survival probability between 2 curves with a p-value of <0.0083 (= 0.05/6) was considered significant. p <0.0001 for patient groups with 1st met (-)/2nd met (-) vs. 1st met (+)/2nd met (-), or 1st met (-)/2nd met (+), or 1st met (+)/2nd met (+), and 1st met (+)/2nd met (-) vs. 1st met (+)/2nd met (+); p = 0.0078 for patient groups with 1st met (-)/2nd met (+) vs. 1st met (+)/2nd met (+); p = 0.222 for patient groups with 1st met (+)/2nd met (-) vs. 1st met (-)/2nd met (+).

Consistent with these results, cumulative incidence function of the same 548 HNSCC patient cohort revealed that the prognosis of HNSCC patients with 1st or 2nd met was significantly worse than patients without nodal metastasis (p <0.0001, Gray’s test with Bonferroni correction) (**Fig 2**). Furthermore, the prognosis was much worse for HNSCC patients with 1st and 2nd mets than patients with a single type of met alone [1st met (+)/2nd met (-), p <0.0001; 1st met (-)/2nd met (+), p = 0.0078].

### Prognostic importance of ENS in 1st and 2nd mets

The predictive ability of ENS in the 1st and 2nd mets for cancer-specific survival of HNSCC patients was investigated in 516 patients with histologically or clinically confirmed ENS-positive or ENS-negative mets. Multivariable Cox proportional hazard regression analysis indicated that ENS in the 1st met [ENS-positive 1st met (+) vs. ENS-positive 1st met (-), adjusted HR = 3.15; 95% CI, 1.40–7.56; p = 0.006] and 2nd met [ENS-positive 2nd met (+) vs. ENS-positive 2nd met (-), adjusted HR = 4.03; 95% CI, 1.41–16.96; p = 0.007] were both significant and independent predictors of poor prognosis, but the 1st met and 2nd met per se were not (**Table 3**).

**Table 3:** Cox proportional hazard regression analysis for the prognostic impact of ENS-positive 1st or 2nd mets on 516 patients (Group II).

	n	hazard ratio	95% CI	p-value
univariate analysis				
primary site				
hypopharynx vs. others	42/474	2.18	1.04–4.10	0.04
1st met				
size/number/location§				
≤3 cm/single/ipsi vs. 1st met (-)	44/391	2.41	1.09–4.77	0.03
3< and ≤6 cm/single/ipsi vs. 1st met (-)	4/391	2.23 x 10^−9^	0–7.38	0.46
≤6 cm/multiple/ipsi vs. 1st met (-)	52/391	2.44	1.19–4.62	0.02
≤6 cm/single or multiple/contra vs. 1st met (-)	20/391	3.47	1.19–8.09	0.03
>6 cm vs. 1st met (-)	5/391	2.21 x 10^−9^	0–3.01	0.25
≤6 cm/multiple/ipsi vs. ≤3 cm/single/ipsi	52/44	1.01	0.42–2.52	0.97
1st met (+) vs. 1st met (-)	125/391	2.35	1.40–3.88	0.002
number of ENS-positive 1st met				
1 vs. 0	39/462	2.99	1.37–5.83	0.008
2 vs. 0	15/462	6.43	2.65–13.35	<0.001
2 vs. 1	15/39	2.15	0.77–5.78	0.14
2/1 vs. 0	54/462	3.91	2.14–6.77	<0.001
2nd met				
number of 2nd met				
1 vs. 0	53/437	4.89	2.69–8.58	<0.001
2 vs. 0	26/437	9.83	4.83–18.79	<0.001
2 vs. 1	26/53	2.01	0.94–4.19	0.07
2/1 vs. 0	79/437	6.09	3.69–10.02	<0.001
number of ENS-positive 2nd met				
1 vs. 0	44/462	7.30	4.18–12.41	<0.001
2 vs. 0	10/462	13.13	4.94–29.28	<0.001
2 vs. 1	10/44	1.80	0.66–4.23	0.23
2/1 vs. 0	54/462	8.08	4.85–13.31	<0.001
multivariable analysis				
primary site				
hypopharynx vs. others	42/474	1.71	0.80–3.34	0.16
1st met				
1st met (+) vs. 1st met (-)	125/391	1.06	0.48–2.09	0.89
ENS-positive 1st met (+) vs. ENS-positive 1st met (-)	54/462	3.15	1.40–7.56	0.006
2nd met				
2nd met (+) vs. 2nd met (-)	79/437	2.01	0.48–5.63	0.29
ENS-positive 2nd met (+) vs. ENS-positive 2nd met (-)	54/462	4.03	1.41–16.96	0.007

Cox proportional hazard regression analysis was performed in 516 HNSCC patients who were selected from the original 548 patients as those having histologically (1st mets) and histologically or clinically (2nd mets) confirmed ENS-positive or -negative mets. 95% CI, 95% confidence interval. Two variables that are separated by ‘vs.’ were compared.

§, Mets were categorized into those that had occurred in the neck sides ipsilateral (ipsi) or contralateral (contra) to the primary lesions.

### Relative risks of ENS in 1st and 2nd mets

**Fig 3:**
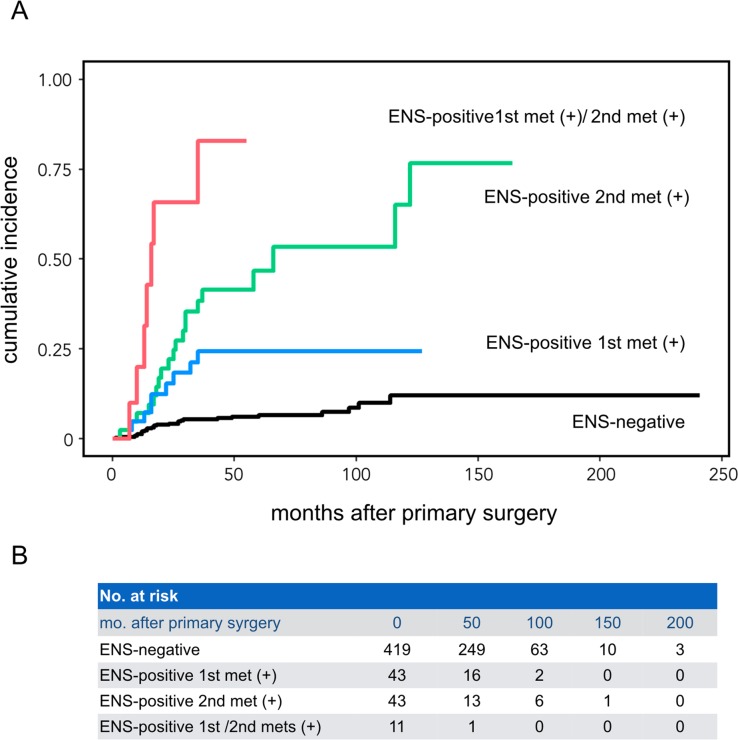
Cumulative incidence function for assessing the influence of ENS on prognoses in 516 HNSCC patients with different ENS-positive met profiles. The number of cases at risk (no. at risk) at each time point (0–200 months) was provided in B. The Bonferroni correction was used to adjust p-values in multiple comparisons in the Gray’s test and a difference in survival probability between 2 curves with a p-value of <0.0083 (= 0.05/6) was considered significant. p <0.0001 for patient groups without ENS (ENS-negative) vs. ENS-positive 1st met (-)/ENS-positive 2nd met (+) [ENS-positive 2nd met (+)], or ENS-positive 1st met (+)/ENS-positive 2nd met (+) [ENS-positive 1st met (+)/2nd met (+)]; p = 0.0003 for ENS-negative patient group vs. ENS-positive 1st met (+)/ENS-positive 2nd met (-) [ENS-positive 1st met (+)]; p = 0.0004 for ENS-positive 1st met (+) patient group vs. ENS-positive 1st met (+)/2nd met (+) patient group; p = 0.0031 for ENS-positive 2nd met (+) group vs. ENS-positive 1st met (+)/2nd met (+) group; and p = 0.052 (not significant) for ENS-positive 1st met (+) group vs. ENS-positive 2nd met (+) group.

Next, we investigated the relative risks of ENS that appeared solely in the 1st or 2nd met, or that appeared in both the 1st and 2nd mets. Cumulative incidence function revealed that the cancer-specific survival of ENS-positive 1st met (+)/ENS-positive 2nd met (-) or ENS-positive 1st met (-)/ENS-positive 2nd met (+) patients was significantly lower than that of patients without ENS (p = 0.0003 and p <0.0001, respectively; Gray’s test with Bonferroni correction) (**Fig 3A and 3B**). However, the difference in survival between the groups with either type of ENS-positive met did not quite reach the significance level (p = 0.052). In contrast, patients with ENS double-positive mets [ENS-positive 1st met (+)/ENS-positive 2nd met (+)] had a significantly worse prognosis than patients with either type of ENS-positive met [p = 0.0004 for vs. ENS-positive 1st met (+)/ENS-positive 2nd met (-), and p = 0.0031 for vs. ENS-positive 1st met (-)/ENS-positive 2nd met (+)].

**Fig 4:**
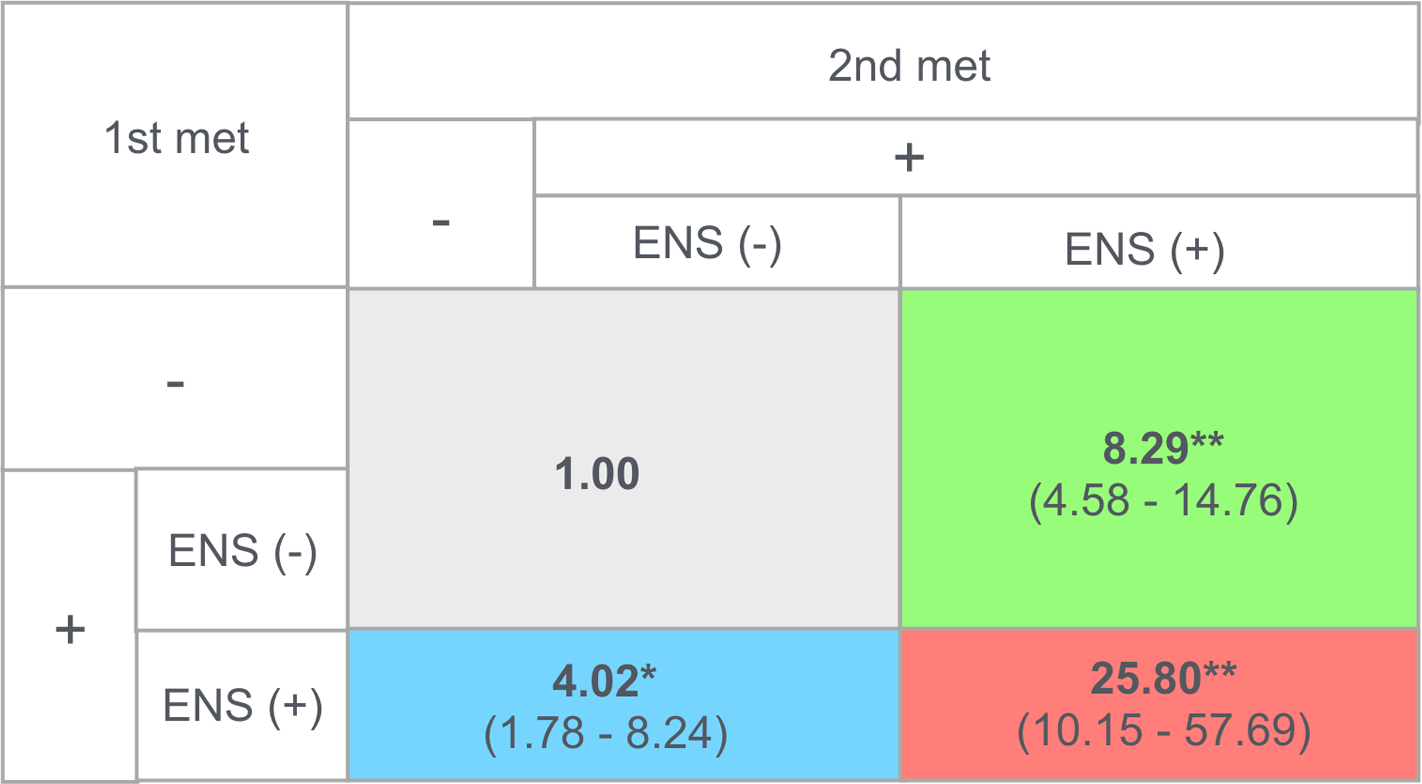
Hazard ratios from Cox proportional hazard analysis. Hazard ratios were calculated with ENS-positive 1st met (-)/ENS-positive 2nd met (-) patient group [including 1st met (-)/2nd met (-), 1st met (+)/ENS-positive 1st met (-)/2nd met (-), and 1st met (-)/2nd met (+)/ENS-positive 2nd met (-) groups] used as a reference (hazard ratio = 1.00) of the other patient groups. *, p = 0.002 for groups with ENS-positive 1st met (-)/ENS-positive 2nd met (-) vs. ENS-positive 1st met (+)/ENS-positive 2nd met (-); **, p <0.001 for groups with ENS-positive 1st met (-)/ENS-positive 2nd met (-) vs. ENS-positive 1st met (-)/ENS-positive 2nd met (+), and ENS-positive 1st met (+)/ENS-positive 2nd met (+) (log-rank test). Numerical data in parentheses indicate 95% confidence intervals.

To further evaluate the relative importance of ENS-positive 1st and 2nd mets as a prognostic variable of HNSCC patients, we determined the relative HRs for patients with various ENS met profiles (**Fig 4**). Cox proportional hazard regression analysis showed that the relative HRs of ENS-positive 1st met (+)/ENS-positive 2nd met (-) and of ENS-positive 1st met (-)/ENS-positive 2nd met (+) patients were 4.02 (95% CI, 1.78–8.24; p = 0.002) and 8.29 (95% CI, 4.58–14.76; p <0.001), respectively. The relative ratio was further increased to 25.80 (95% CI, 10.15–57.69; p <0.001) in patients with ENS double-positive mets.

### Influence of 1st met and/or ND history for ENS-positive 2nd met development

**Fig 5:**
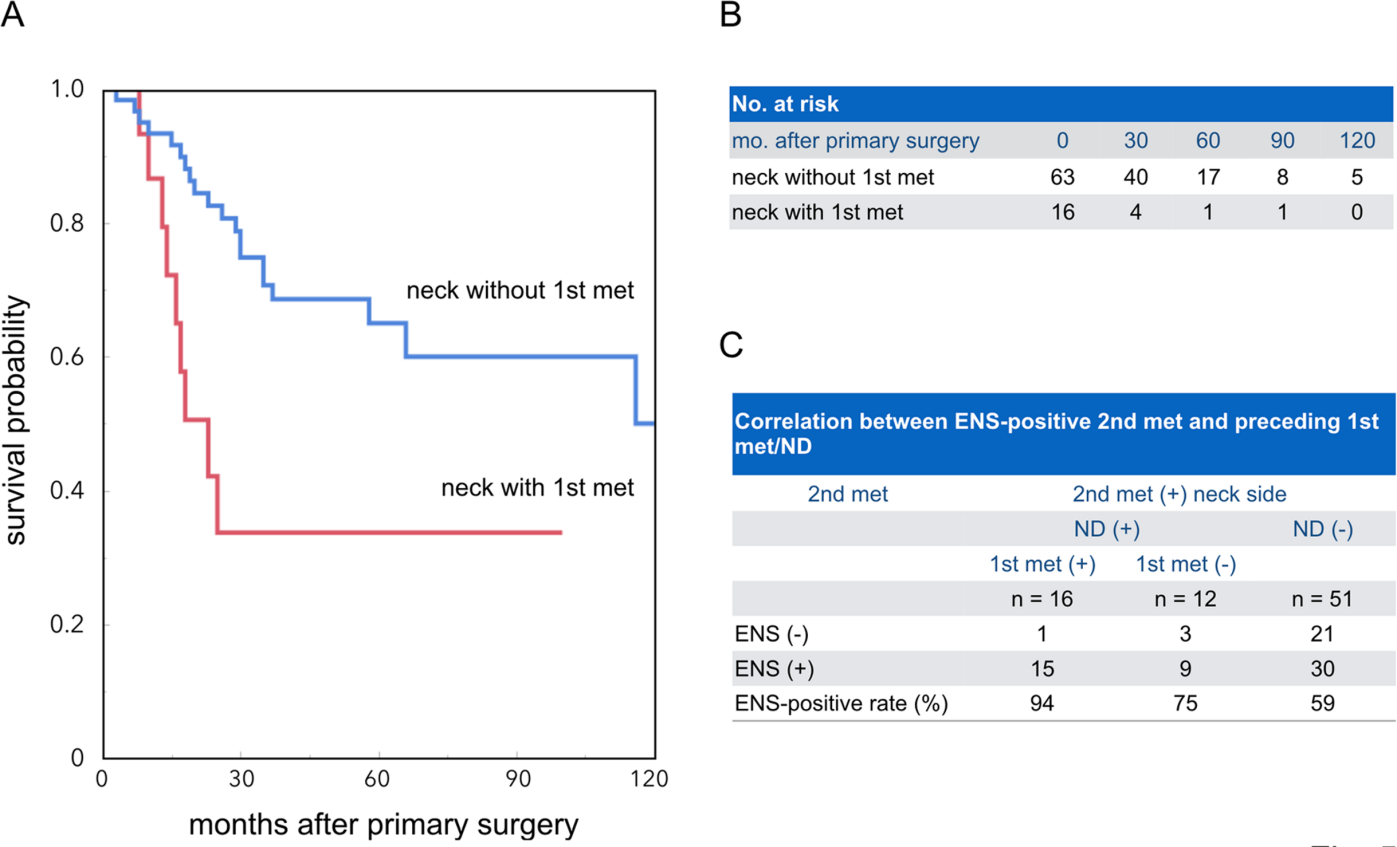
Influence of pre-existing 1st met and history of ND in the same neck side of 2nd met. **A**, **B**, Kaplan-Meier analysis in 95 HNSCC patients with 2nd mets. The number of cases at risk (no. at risk) at each time point (0–120 months) was provided in B. p <0.003 for patient groups with (neck with 1st met) vs. without (neck without 1st met) preceding 1st met and ND in the same neck side of 2nd met (log-rank test). **C**, Correlations between ENS in 2nd mets and 1st met and/or ND history in the same neck sides of 79 HNSCC patients with 2nd met. ENS-positive rates were calculated for patient groups with different profiles of 1st met and ND history, including 1st met (+)/ND (+), 1st met (-)/ND (+), and 1st met (-)/ND (-). p = 0.008 for different groups (Cochran-Armitage test).

Lastly, we asked whether the history of 1st met and ND can affect the prognosis of patients with 2nd met. To this end, we compared the survival probability between patients with or without a history of 1st met in a patient group having histologically and/or clinically confirmed 2nd met(s) (n = 95, see Group 1 in **Fig 1**). This patient subcohort did not include any competing risks. Therefore, we used Kaplan-Meier analysis instead of the cumulative incidence function for assessing the survival probability. The analysis indicated that the survival probability of patients with 2nd met in the neck side where a preceding 1st met had occurred and the subsequent ND performed was significantly lower than that of patients with 2nd met in the neck without any history of 1st met (p = 0.003, **Fig 5A and 5B**).

A further investigation using a limited study population of patients with ENS-positive or ENS-negative 2nd met (n = 79, see Group II in **Fig 1**) showed significantly different occurrence rates of ENS-positive 2nd met in the neck with preceding 1st met and ND, from those with ND history alone, and those without any history of 1st met or ND (p = 0.008, Cochran-Armitage test, **Fig 5C**).

## Discussion

In the present study, we investigated the relative risks of ENS in the 1st and 2nd mets as prognostic factors in HNSCC patients who underwent surgical excision of the primary tumors. We found that ENS in the 1st and 2nd mets were the dominant risk factors in patients over other tested factors, including age, sex, primary site, ND history, and the profiles (numbers and locations in the neck) of 1st and 2nd mets. An ENS-positive 2nd met carried a similar prognostic risk level as ENS-positive 1st met; however, the double-positive ENS in the 1st and 2nd mets in the same neck indicated a much worse prognosis than the presence of either type of ENS-positive met. Furthermore, a preceding 1st met with an ND history was a risk factor of ENS-positive 2nd met in the same neck side.

As mentioned in the methods section, the 2nd met was defined as one that occurred in the absence of primary tumor recurrence and distant metastasis. Therefore, patients having such histories were tentatively categorized into the 2nd met (-) group in the present study, and the assessment for risk factors was conducted under the assumption that the primary lesions were kept under control during the periods from the surgery up to the development of 2nd met. However, in previous studies, the prognostic outcomes had been greatly affected by the conditions of the primary lesions [1, 2]. Thus, the present results suggest that the prognosis of HNSCC patients who had undergone excisions of the primary tumors can exclusively depend on the ENS profiles of 1st and 2nd mets provided the primary lesions are controlled.

The prognostic importance of ENS has been controversial for patients with HNSCC. For example, de Juan et al. showed that knowing the the presence or absence of ENS in the ND improved the predictive ability of tumor-node-metastasis (TNM) classification in HNSCC patients [8]. However, Sinha et al. did not find any evidence that ENS could be predictive in patients with p16-positive oropharyngeal SCC [9]. More recently, Wreesmann et al. has shown that prognosis depended upon the extent of ENS beyond the nodal capsules, and that the prognosis of oral SCC patients with minor (≤1.7 mm beyond the capsule) ENS-positive node was not different from that of patients without ENS-positive nodes [10]. The heterogeneity of the patient populations in terms of HNSCC primary sites may be another factor contributing to the observed discrepancies. In the present study, we used patients with a relatively wide spectrum of HNSCC primary sites. However, Shingaki et al. analyzed the risk factors in a more homogeneous patient population (oral cavity SCC) to show that ENS was a significant predictor for patient survival, albeit with borderline significance in multivariable analysis [11]. It is plausible that SCCs in the different primary tumor sites grow, invade the surrounding organs, and metastasize to regional lymph nodes in different ways. Consistent with this idea, it is well known that the hypopharynx has a rich mucosal and submucosal lymphatic capillary network [12]. Accordingly, hypopharyngeal SCC has a high propensity for metastasizing to regional lymph nodes. In the present study, the primary site (hypopharynx and oral cavity) was found to be an important factor for the occurrence of ENS-positive 2nd mets, but was not an independent predictor of poor prognosis. In this regard, the recently updated criteria for the N-classification of oral SCC incorporated ENS as a critical item, but not for the classification of other HNSCC subtypes [13].

Multiple occurrences of ENS-positive 1st mets have been indicated as higher-risk factor in patients with HNSCC compared to a single occurrence of an ENS-positive met [14, 15]. However, the present study did not support this concept. Instead, the occurrence of ENS-positive met at different stages (i.e., 1st and 2nd mets) in a single HNSCC patient significantly decreased the cancer-specific survival. The present study demonstrated that ENS in the 1st and 2nd mets was the dominant risk factors to the HNSCC patient, and that even the profiles of 1st and 2nd mets did not independently and significantly affect the patient outcomes. However, the present results should be carefully interpreted, considering that we did not verify (1) the influence of the different treatment types performed, (2) p16 expression, a prognostic marker, for predicting prognosis of oropharyngeal cancer, and (3) the T classification specific for different primary sites as a possible prognostic factor [8, 9]. The notion that ENS is the dominant prognostic factor could be verified by assessing the prognostic outcomes of oral SCC patients who are categorized into subgroups according to the new N classification system, and then analyzing the ENS profiles in the 1st and 2nd mets within each group.

It should also be noted that the preexisting 1st met with ND history could be a risk factor in patients with ENS-positive 2nd met. The significantly higher rates of ENS-positive 2nd met in the patient group with a preexisting 1st met(s) and ND history within the same neck sides than in the group without these neck profiles (94% vs. 62%) suggest that the neck with these histories might be prone to developing ENS-positive mets. At this time, it is not well understood how ENS occurs in mets. Sumi and Nakamura showed that fibrotic foci were found in the immediate vicinity of ruptured nodal capsules of SCC nodes in the neck, and that those areas often contained cancer cell nests [16]. Intratumoral fibrotic foci are thought to indicate hypoxic areas [17]. Considering the current consensus that intratumoral hypoxic conditions could promote aggressive tumor growth [18], detection of intranodal hypoxia might facilitate the early and precise detection of ENS-positive mets with capsular ruptures. In terms of molecular mechanisms leading to ENS, recent studies have evaluated a possible role for lysyl oxidase in tumor hypoxia [19]. Furthermore, a close relationship between lysyl oxidases and ErbB2 signaling has been implicated as a molecular mechanism of nodal rupture [20, 21].

This study is partly limited by its retrospective nature. First, surgical excision of the primary lesions without preoperative radiotherapy was one of the inclusion criteria, but protocols for post-operative chemoradiotherapy (radiation doses and chemotherapy protocols) and ND types (radical, selective, or extended radical) were not fully controlled for in the present study. Second, all the 1st mets were histologically confirmed, but 2nd mets were clinically diagnosed on CT/MR images in 16 of the 79 patients. Although CT/MR imaging diagnosis of mets with ENS produced acceptable results [16, 22, 23], a very small number of false-positive and false-negative results could not be avoided. Third, we did not assess T classification for predicting patient prognosis. We studied a HNSCC patient cohort with widely varying primary sites. The T classification is defined differently for HNSCCs in different primary sites. Therefore, the importance of T classification should be separately evaluated in HNSCC patients with different primary sites, even if it means decreasing the cohort size [24].
